# Necrology

**Published:** 1901-02

**Authors:** 


### NECROLOGY.

AT Pottstown, Pa., January 10, 1901, Dr. Nathan Evans Reinhart, aged sixty-four years. Dr. Reinhart was an active practitioner until the time of his death, although a sufferer for many months from a cancer of the face and throat; an earnest and interested member of the Pennsylvania State Veterinary Medical Association for many years; a man of kind disposition and affable temperament, and highly respected by all who were favored with his acquaintance.

